# An artificial neural network for membrane-bound catechol-*O*-methyltransferase biosynthesis with *Pichia pastoris* methanol-induced cultures

**DOI:** 10.1186/s12934-015-0304-7

**Published:** 2015-08-07

**Authors:** Augusto Q Pedro, Luís M Martins, João M L Dias, Maria J Bonifácio, João A Queiroz, Luís A Passarinha

**Affiliations:** CICS-UBI, Centro de Investigação em Ciências da Saúde, Universidade da Beira Interior, Avenida Infante D. Henrique, 6201-001 Covilhã, Portugal; Department of Biochemistry, Cambridge System Biology Centre, University of Cambridge, Sanger Building, 80 Tennis Court Road, Cambridge, CB2 1GA UK; Departamento de Investigação e Desenvolvimento, Bial, 4745-457 São Mamede do Coronado, Portugal

**Keywords:** Catechol-*O*-methyltransferase, Artificial neural network, Bioreactor, *Pichia pastoris*, DMSO, Alcohol oxidase, Membrane protein

## Abstract

**Background:**

Membrane proteins are important drug targets in many human diseases and gathering structural information regarding these proteins encourages the pharmaceutical industry to develop new molecules using structure-based drug design studies. Specifically, membrane-bound catechol-*O*-methyltransferase (MBCOMT) is an integral membrane protein that catalyzes the methylation of catechol substrates and has been linked to several diseases such as Parkinson’s disease and Schizophrenia. Thereby, improvements in the clinical outcome of the therapy to these diseases may come from structure-based drug design where reaching MBCOMT samples in milligram quantities are crucial for acquiring structural information regarding this target protein. Therefore, the main aim of this work was to optimize the temperature, dimethylsulfoxide (DMSO) concentration and the methanol flow-rate for the biosynthesis of recombinant MBCOMT by *Pichia pastoris* bioreactor methanol-induced cultures using artificial neural networks (ANN).

**Results:**

The optimization trials intended to evaluate MBCOMT expression by *P. pastoris* bioreactor cultures led to the development of a first standard strategy for MBCOMT bioreactor biosynthesis with a batch growth on glycerol until the dissolved oxygen spike, 3 h of glycerol feeding and 12 h of methanol induction. The ANN modeling of the aforementioned fermentation parameters predicted a maximum MBCOMT specific activity of 384.8 nmol/h/mg of protein at 30°C, 2.9 mL/L/H methanol constant flow-rate and with the addition of 6% (v/v) DMSO with almost 90% of healthy cells at the end of the induction phase. These results allowed an improvement of MBCOMT specific activity of 6.4-fold in comparison to that from the small-scale biosynthesis in baffled shake-flasks.

**Conclusions:**

The ANN model was able to describe the effects of temperature, DMSO concentration and methanol flow-rate on MBCOMT specific activity, as shown by the good fitness between predicted and observed values. This experimental procedure highlights the potential role of chemical chaperones such as DMSO in improving yields of recombinant membrane proteins with a different topology than G-coupled receptors. Finally, the proposed ANN shows that the manipulation of classic fermentation parameters coupled with the addition of specific molecules can open and reinforce new perspectives in the optimization of *P. pastoris* bioprocesses for membrane proteins biosynthesis.

**Electronic supplementary material:**

The online version of this article (doi:10.1186/s12934-015-0304-7) contains supplementary material, which is available to authorized users.

## Background

Membrane proteins (MP) are central to many cellular processes: they are involved in the uptake and export of diverse charged and uncharged molecules, as well as mediating the interaction of cells with their environment [1]. As a consequence, they are of prime importance as drug targets to the pharmaceutical industry [1]. Catechol-*O*-methyltransferase (COMT, EC 2.1.1.6) is a magnesium-dependent enzyme that catalyzes the methylation of catechol substrates using *S*-adenosyl-l-methionine (SAM) as a methyl donor and yielding, as reaction products, the *O*-methylated catechol and *S*-adenosyl-l-homocysteine [2]. In humans, COMT appears as two molecular forms, a soluble and a membrane-bound isoform (MBCOMT) that is found mainly associated with the rough endoplasmic reticulum membrane [2]. Specifically, SCOMT is a nonglycosylated protein containing 221 amino acid residues and a molecular weight of 24.7 kDa while MBCOMT has an additional peptide in its amino terminal of 50 amino acid residues and a molecular weight of 30 kDa [2].This extra peptide contains a stretch of 21 hydrophobic amino acid residues that constitute the membrane anchor region [2]. In fact, MBCOMT is an integral membrane protein with the catalytic portion of the enzyme oriented toward the cytoplasmic side of the membrane [2]. Recently, MBCOMT has gained a major importance as therapeutic target due to its high abundance in human brain and its higher affinity for catechol substrates when compared to soluble isoform [2]. During the last decades, COMT has been implicated in several diseases such as cardiovascular diseases [3], estrogen-induced cancers [4] and neurologic disorders [2]. Specifically, the best documented is the important role that COMT plays in Parkinson’s disease whose most effective treatment remains the dopamine replacement therapy with levodopa together with an inhibitor of aromatic amino acid decarboxylase and a COMT inhibitor [2]. Therefore, it becomes clear the importance of developing new and more effective drugs for COMT inhibition in which structure-based drug design can play an important role in this process. However, in order to structurally and functionally characterize a MP, a stable active sample is required, meaning the requirement for a regular supply of milligram quantities of purified MP [1]. The foremost requirements associated with the majority of biophysical techniques emphasize the importance of developing new systems capable of delivery biologically active MBCOMT in higher quantities from high cell-density cultures. Around the mid of the twentieth century, bacteria and filamentous fungi have taken over the lead role in the development of bioprocesses [5]. However, novel developments of recombinant protein production, metabolic engineering and systems biology open a range of new applications of yeasts in the upstream stage of a bioprocess [5]. In fact, over the last two decades, the methylotrophic *Pichia pastoris* (*P. pastoris*) has been established as one of the most frequently used expression systems for recombinant protein production [6]. The benefits of this system include growth up to high cell densities quantity on defined minimal medium, high expression level of heterologous proteins, typical eukaryotic post-translational modifications, efficient secretion of extracellular proteins and the presence of the efficient methanol-inducible promoter from alcohol oxidase I gene (AOX) [7, 8]. Moreover, the *P. pastoris* preference for respiratory rather than fermentative metabolism, even at high cell density processes, prevents the accumulation of secondary metabolites such as ethanol and acetic acid [7]. Finally, following the recognition of *P. pastoris* as a GRAS organism by FDA in 2006 [6], the importance of this host as a platform for biopharmaceuticals production is highlighted. Upon the design of a bioprocess for recombinant protein production in *P. pastoris* under the control of the AOX promoter, a key step is the optimization of the induction phase since it will directly impact on the yield of the process [9]. Over the past few years, many efforts have allowed relevant advances in the development of *P. pastoris* for the production of MP where significant achievements were made in order to improve yield and proper folding of these target proteins [10]. Specifically, chemical chaperones such as dimethylsulfoxide (DMSO) have been shown to increase the expression of different G protein-coupled receptors such as the human neuromedin U subtype II receptor [11], the human adenosine A_2A_ receptor or the human β2-adrenergic receptor [12], mostly due to the up-regulation of the expression of genes involved in membrane lipid components [10, 13]. In addition, it was also reported that lowering the culture temperature from 30 to 20°C also leads to an improvement of the expression of MP, possibly because it slows down protein production, not overloading the translocation machinery, protein processing or intracellular trafficking [13]. Finally, while the methanol feeding strategy is one of the most important factors for maximizing heterologous protein expression, the methanol induction phase may also depend on other operational conditions (temperature, pH and culture medium), phenotype and specific characteristics of the heterologous protein produced [14]. In general, the traditional optimization method, commonly called “one factor/variable at a time”, consists in varying one factor while keeping the others constant [15, 16] and is extremely time-consuming requiring a large number of experiments [15]. In alternative, statistical experimental designs have been widely used and they can be applied at distinct phases of an optimization process, either for screening experiments or for searching for the optimal conditions for targeted response(s) [17]. Overall, response surface methodology (RSM), which includes factorial design and regression analysis, seeks to identify and optimize significant factors to maximize the response [18]. On the other hand, artificial neural networks (ANN) allow estimating relationships between one or more input and one or more output (also called responses) [16]. In general, ANNs are greater and more accurate modeling techniques when compared with RSM since they can cope with nonlinearities among the factor in the prediction of a given response [18]. Indeed, ANNs coupled with design of experiments have been successfully applied in diverse areas such as the optimization of the culture conditions [16, 18], pharmaceutics [19] or chromatography [15, 20].

The main aim of this work was to optimize the induction phase for recombinant MBCOMT production by *P. pastoris* X33 Mut^+^ cultures in bioreactor applying central composite design (CCD) and ANNs.

## Results and discussion

The structural and functional characterization of a MP depends on the production of a sufficient amount of active protein, meaning a regular supply of milligram quantities of the target enzyme [1]. Therefore, to fulfill this requirement, in this work and for the first time the biosynthesis of MBCOMT by *P. pastoris* bioreactor cultures is reported. Initially, in order to select the most appropriated *P. pastoris* strain for MBCOMT biosynthesis, trials at a small-scale in baffled shake-flasks were carried out. Then, a three-stage bioprocess for the biosynthesis of the target protein by *P. pastoris* bioreactor cultures was implemented and the lengths of the glycerol fed-batch and the methanol induction phases were optimized.

Moreover, after selecting a set of independent variables associated with the methanol induction phase that greatly influence the levels of the MBCOMT, ANN modeling was carried out in order to maximize the biological activity of the target protein. The massic and volumetric productivities were not incorporated as an output since the values of those parameters are in strictly dependence on MBCOMT biological activity [18]. Also, the biomass levels were evaluated in all assays performed in this work but were not considered in the optimization and validation procedures as an output, since higher biomass levels not always lead to higher mass productivities of the target protein.

### Small-scale MBCOMT biosynthesis in *P. pastoris*

Membrane-bound catechol-*O*-methyltransferase biosynthesis was initially carried out in shake-flasks containing BMGH medium using a Mut^+^ (X33) and a Mut^S^ (KM71H) *P. pastoris* strains [21]. Sometimes, an increase in the number of the heterologous gene can possibly lead to an increase in transcription and translation rate of the desired gene [22]. In fact, although opposite results had already been published, there are several examples including the mouse epidermal growth factor or miniproinsulin in which higher target gene copy numbers lead to higher titers for *P. pastoris* bioprocesses driven by AOX1 promoter [22]. Therefore, upon the transformation procedure with the target recombinant plasmid, clones from both strains in study were isolated from plates containing high zeocin concentrations (2 mg/mL). Following the isolation of these clones from both strains, it was determined the target gene copy number that was integrated in each strain. Therefore, using the method previously reported by Nordén and collaborators [23] that takes advantage of the fact that part of the plasmid pPICZ α, namely the AOX1 TT region is incorporated in the *P. pastoris* genome together with the gene to be expressed. In particular, for the X33 strain, the primer efficiencies were 1.88 and 1.87, respectively for the AOX1 TT and AOX2 PROM primer pairs. Similarly, for the KM71H strain, the primer efficiencies were 1.91 and 1.94, respectively, for the AOX1 TT and AOX2 PROM primer pairs. Finally, according the equation described in the “Methods”, the target gene copy number introduced in each recombinant strain was determined and it was found that X33-PICZα-MBCOMT had nine copies of the target plasmid while the KM71H-PICZα-MBCOMT had ten copies. In fact, Nordén and coworkers [23] reported with the aquaporins that colonies isolated from 0.5 mg/mL zeocin could harbor from 4 to 15 plasmids while from 1 mg/mL, as many as 17 heterologous DNA sequences can be incorporated. Therefore, although the isolation of clones from plates containing higher antibiotic concentrations doesn’t exclude completely the occurrence of false positives, the values here reported (9 and 10 copies for the X33 and KM71H strains, respectively) are in the same order of magnitude. Then, small-scale fermentation trials were carried out using 0.5% (v/v) methanol and higher biomass levels were detected for the X33 strain (OD_600_ = 7.5) when compared with those obtained for the KM71H strain (OD_600_ = 1.8). Similarly, the target enzyme recovered from the X33 strain presented higher biological activity (60.25 nmol/h/mg) in comparison to KM71H cells (25.77 nmol/h/mg of protein) [21]. On the other hand, when the methanol concentration is lowered from 1 to 0.25% (v/v), similar values for MBCOMT biological activity were obtained for the X33 (61.73 nmol/h/mg of protein) and the KM71H (60.62 nmol/h/mg of protein) strains [21]. Specifically, we believe that the observed differences in these two strains concerning their performance in MBCOMT biosynthesis seem to be associated with the methanol concentration used for induction and not for example with the target gene copy number inserted in the genome since it is similar.

The value previously reported [21] with both *P. pastoris* strains for MBCOMT biological activity is higher than those previously reported by our research group using *Brevibacillus choshinensis* as the expression system (48.07 nmol/h/mg of protein) [24]. In general, for intracellular expression, it has been reported that it is preferable use Mut^S^ over Mut^+^*P. pastoris* strains because of increased specific yield of heterologous protein [25]. However, as previously reported by Maurer and collaborators, the volumetric productivity QP is the most plausible target for optimization in fed-batch processes [26]. Therefore, since the main aim of this work was to maximize MBCOMT expression irrespective the biomass levels, *P. pastoris* Mut^+^ X33 was chosen for further bioreactor trials since regardless the methanol concentration used, the expression levels of the target protein were the highest obtained and they didn’t significantly change when different methanol concentrations are applied.

### MBCOMT biosynthesis from methanol-induced *Pichia pastoris* bioreactor cultures

Membrane-bound catechol-*O*-methyltransferase biosynthesis was carried out in mini-bioreactors (working volume 0.25 L) in modified basal salts medium (BSM) containing 4.35 mL/L trace metal solution (SMT) [27] and the pH was adjusted to 4.7 in order to minimize precipitation and, consequently, undesired operational problems such as starvation of nutrients and optical densities measurement interferences [14]. *P. pastoris* cultivations in bioreactor were initiated with a glycerol batch phase (30 g/L glycerol) that ends when glycerol was depleted, indicated by a sharp increase in the dissolved oxygen (DO) [14]. After this stage, a fed-batch growth on glycerol [50% (v/v) at 18.54 mL/L/H] during different periods was employed, followed by the methanol induction phase where *P. pastoris* was cultivated on a methanol fed-batch mode. In order to promote the derepression of the AOX promoter prior to induction, 1 h before starting the induction phase, methanol was added to the reaction vessel at the flow-rate later employed in the methanol fed-batch phase.

**Fig. 1:**
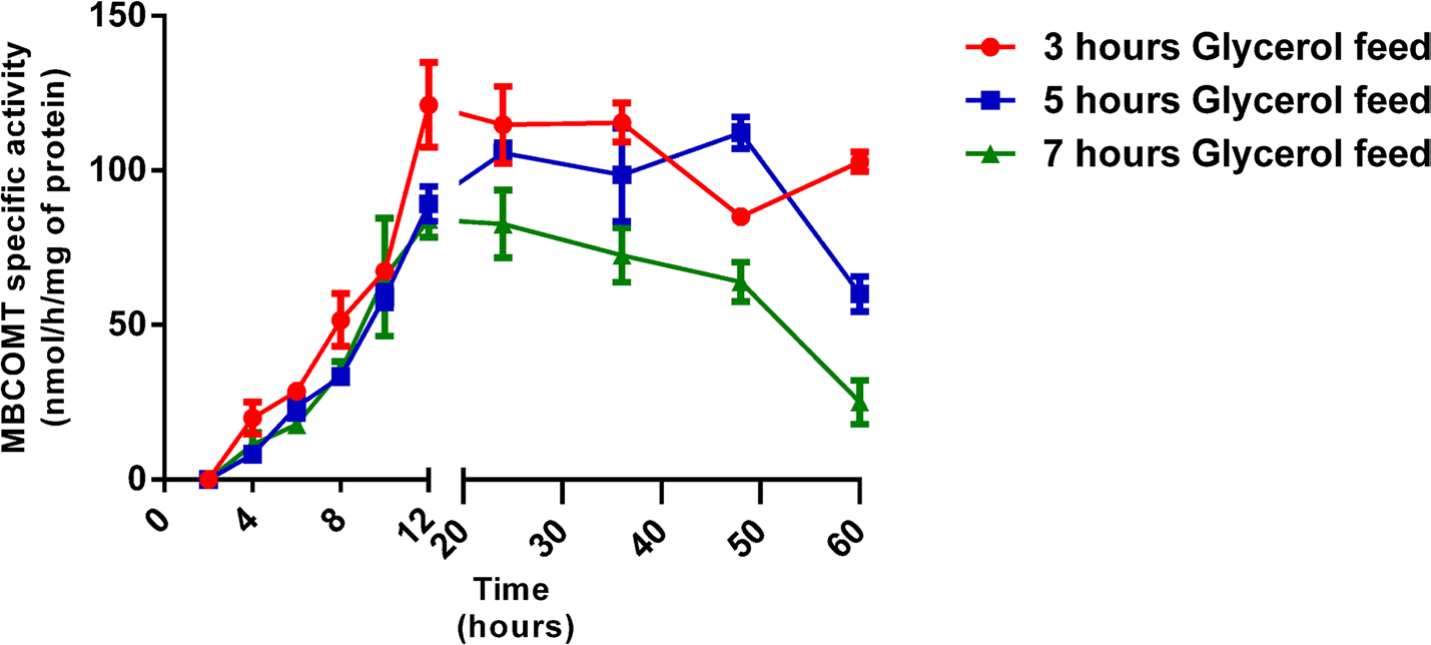
Typical time profile of MBCOMT specific activity (nmol/h/mg of protein) obtained by *P. pastoris* bioreactor cultures using different periods of the glycerol fed-batch phase with a methanol constant feed flow-rate at 3.6 mL/L/H (each value represents the mean of three independent samples).

Preliminary trials were carried out in order to analyze the optimal period of the glycerol fed-batch phase as well as the optimal duration of the methanol induction phase that maximize MBCOMT expression. Therefore, keeping constant the methanol flow-rate (3.6 mL/L/H) in the induction phase, assays with 3, 5 or 7 h glycerol fed-batch phase were carried out. Methanol induction phase was maintained during 60 h and samples were collected with an interval of 2 h until 12 h and then every 12 h to follow the MBCOMT expression profile. As depicted in Fig. 1, the highest MBCOMT biological activity levels were detected when a 3 h period was applied in the glycerol fed-batch phase. In addition, concerning to the methanol induction phase, MBCOMT achieved a maximum expression of 121.0 nmol/h/mg of protein at 12 h of induction, what led us to assume a 3 h glycerol fed-batch period and a 12 h induction period for further experiments. In fact, a shorter induction period can be greatly advantageous over other previously reported strategies [27, 28] where induction usually takes more than 48 h, being more time-consuming and laborious. Moreover, the shorter induction period allows terminating the fermentation before a decrease in the cell’s physiological activity is observed [29].

**Fig. 2:**
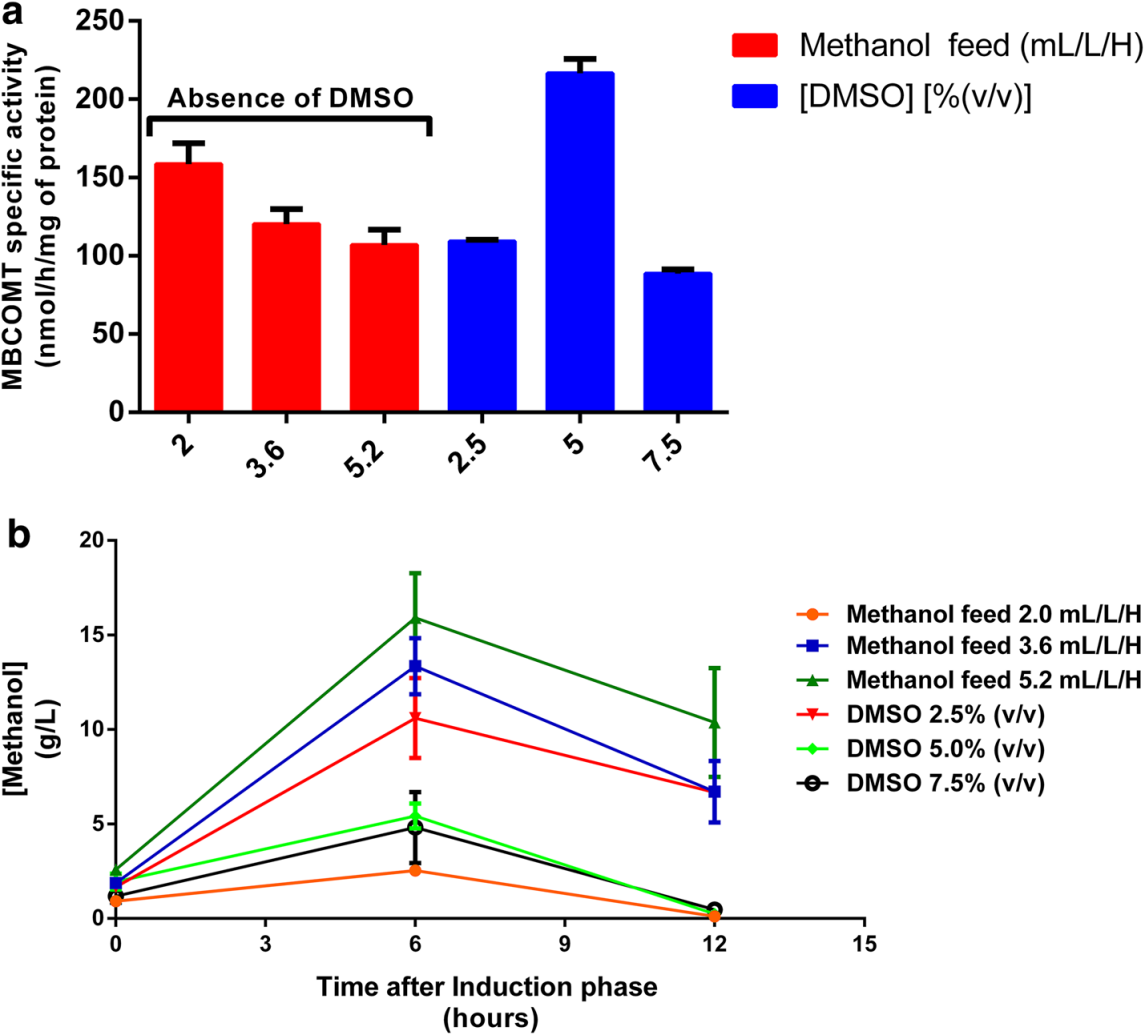
**a** Analysis of different methanol flow-rates (without the addition of DMSO) and different DMSO concentrations (keeping constant the methanol flow-rate at 3.6 mL/L/H) on MBCOMT specific activity (nmol/h/mg of protein) obtained by *P. pastoris* bioreactor cultures. **b** Time course analysis of the methanol levels in the above mentioned assays measured by HPLC-RID. In both experiments, a 3-h period of the glycerol fed-batch was applied and induction was conducted during 12 h (each value represents the mean of three independent samples).

Following these findings, we evaluated if the expression of the target protein was significantly affected by the methanol constant flow-rate as well as the addition of the chemical chaperone DMSO that has been described to increase the expression levels of some MP [11–13, 30, 31]. Therefore, keeping constant the operational parameters previously optimized, distinct assays were performed: with different methanol constant flow rates at 2, 3.6 and 5.2 mL/L/H while others were performed maintaining the methanol flow-rate at 3.6 mL/L/H and changing the DMSO concentration [2.5, 5 and 7.5% (v/v)] in the culture according to what previously described [11–13, 30]. As demonstrated in Fig. 2a, for the lowest methanol constant flow-rate (2 mL/L/H), a highest MBCOMT expression level of 158 nmol/h/mg were obtained, contrasting with 120 and 107 nmol/h/mg for 3.6 and 5.2 mL/L/H, respectively. Also, the methanol and the biomass levels at distinct stages of the induction phase were quantified in these assays, as depicted in Fig. 2b and Table 1, respectively. In general, for the different methanol flow-rates applied, the methanol levels increase from 0 to 6 h and then they decrease until the end of the induction phase. At the early stage of the induction phase, methanol doesn’t seem to be consumed in a large extent since *P. pastoris* cells may be going through a transition period where they stop consuming glycerol and start to oxidize methanol. Nevertheless, it is possible to observe that for methanol constant-flow rates of 3.6 and 5.2 mL/L/H, the concentration of methanol in the culture broth is higher (near 10 and 12.5 g/L respectively) at 6 h of induction when compared with the lowest flow-rate employed (1 g/L). Therefore, it is feasible to assume that using a lower flow rate (2 mL/L/H) allows the establishment of an appropriated balance between activation of the AOX promoter and, consequently, production of the target enzyme and accumulation of methanol in the culture medium that can be responsible for the undesired toxicity, as it may be happening for 3.6 and 5.2 mL/L/H [14]. Moreover, an optimal ratio of methanol to cell concentration should be applied [32], otherwise high methanol feeding rates stress the cell machinery and negatively affect the process performance [32, 33].

**Table 1:** Time course profile of the biomass levels (measured as OD_600 nm_) obtained in the trials where the methanol constant feed flow-rate (2, 3.6 and 5.2 mL/L/H) and the DMSO levels added to the culture were changed, in accordance with the results shown in Fig. 2b

Time after induction phase (h)	Optical density measurements at 600 nm					
	Methanol constant feed flow-rate			DMSO concentration		
	2 mL/L/H	3.6 mL/L/H	5.2 mL/L/H	2.5% (v/v)	5% (v/v)	7.5% (v/v)
3	111.75 ± 1.23	105.19 ± 5.57	116.75 ± 4.42	112.75 ± 4.95	113.44 ± 4.33	104.88 ± 2.47
9	110.32 ± 2.38	106.88 ± 7.95	110.5 ± 3.36	110.19 ± 2.21	114.43 ± 1.17	113.44 ± 0.27
15	111.31 ± 4.68	111.5 ± 9.02	117.38 ± 2.47	116.31 ± 2.21	132.00 ± 7.07	115.06 ± 3.62

On the other hand, when different DMSO concentrations were added to the *P. pastoris* cultures, the highest MBCOMT biosynthesis of 216 nmol/h/mg was detected for 5% (v/v), which represents an increase of 1.8-fold when compared with the control (without DMSO). Again, the methanol levels were also quantified in these trials and interestingly, its time course profile with the addition of 5% (v/v) DMSO conducted with 3.6 mL/L/H of methanol resembles the profile previously obtained for the 2 mL/L/H methanol flow rate and not the 3.6 mL/L/H. Following these results, it is reasonable to think that adjusting the DMSO concentration to the cell needs, the methanol is more efficiently used what, in a last analysis, leads to an increase in the biosynthesis of the target protein.

The addition of 5% (v/v) DMSO proved to have a positive effect on the expression of this particular MP, has been demonstrated previously for G protein-coupled receptors by other authors [11–13, 30, 31]. Although the mechanism by which DMSO increases MP expression is not yet fully understood, Murata and collaborators showed that DMSO induces membrane proliferation through the increase of the phospholipid content within *Saccharomyces cerevisiae* cells [34]. On the other hand, it was also reported that DMSO possess antioxidant properties, preventing protein oxidation (increase in protein carbonyl content and decrease in free thiol content) in rat brain homogenates induced by ferrous chloride/hydrogen peroxide [35]. Therefore, it is likely that the benefits of using DMSO on the expression of membrane proteins can be associated with the induction of membrane proliferation or with the reduction of protein oxidation or a combination of both. Moreover, despite the optimal temperature for growth and production of proteins in *P. pastoris* is 30°C [14], some authors claim that working at lower temperatures (until 20 to 25°C) may improve the target protein biosynthesis [36], lower cell lysis [37] and decrease the proteolytic activity [38]. Therefore, in this work, the temperature was also included as an independent process parameter to optimize MBCOMT biosynthesis from *P. pastoris* and the ranges (20, 25 and 30°C) were selected according to what has been reported in the literature [14, 37].

**Fig. 3:**
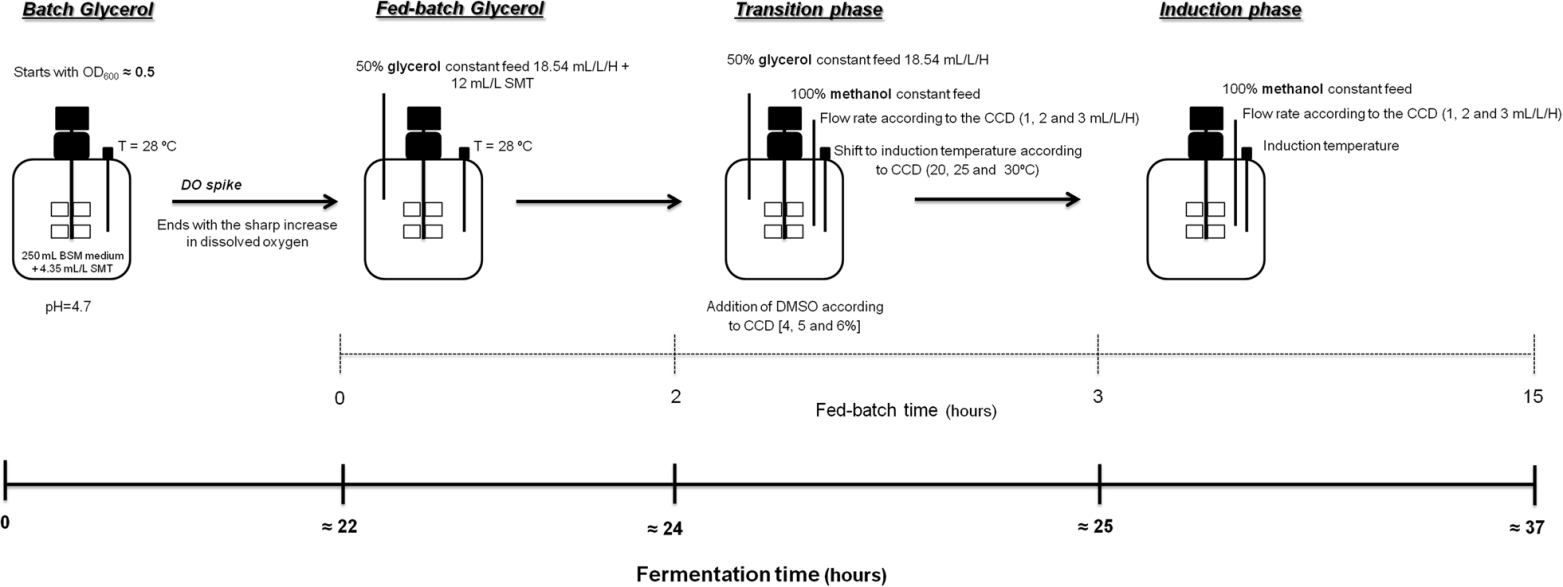
Structure of the optimized four-stage bioprocess implemented in this work for recombinant MBCOMT biosynthesis by *P. pastoris* bioreactor cultures.

According to the results reported in this section and the synergy observed between methanol flow rate and DMSO concentration in the culture broth, the most appropriated ranges of the independent variables selected for performing the experimental design were defined, as shown in Table 2. Finally, a summary of the optimized conditions for the expression of MBCOMT from *P. pastoris* bioreactor methanol-induced cultures is presented in Fig. 3 where the ranges of the independent variables selected for the ANN modeling are presented as well as the major experimental conditions selected.

**Table 2:** Coded levels used for temperature, methanol constant feed flow-rate and DMSO in the CCD

Input variables	Coded levels		
	−1	0	1
Temperature (°C)	20	25	30
Methanol constant feed rate (mL/h/L)	1	2	3
DMSO [% (v/v)]	4	5	6

### Experimental design and artificial neural network modeling

A set of 17 experiments defined by CCD for optimization of the induction phase for maximizing MBCOMT biosynthesis in *P. pastoris* culture are listed in Tables 2 and 3. In general, lower MBCOMT biological activity levels were detected when the input variables defined in CCD were at the lowest levels. Specifically, MBCOMT biosynthesis is maximized at higher methanol constant-flow rate concentrations and when the concentration of DMSO added is higher. On the other hand, an increased in the induction temperature coupled to an increase in the other input variables also lead to an increase in biologically active MBCOMT expression. According to the ANN modeling results in calibration dataset (DoE experiments) (Table 3), the predicted maximum for MBCOMT specific activity (384.8 nmol/h/mg of protein) was achieved at 30°C, 2.9 mL/L/H methanol constant flow-rate and with the addition of 6% (v/v) DMSO. In general, as previously demonstrated for others MP [11–13, 30, 31], the addition of DMSO to the culture proved to have a positive effect on MBCOMT expression since over the model optimization the maximum target protein specific activity is achieved at higher DMSO concentrations. In addition, the output seems to be maximized when the methanol constant flow-rate and the induction temperature are close to the upper values defined in the CCD. This can be explained by the increase in the biomass levels (data not shown) caused by the increase in the temperature and, since there is more methanol that is being oxidized by the AOX promoter, the supply of inducer needs to be higher in order to maintain induction. An ANN model was developed in order to optimize the induction phase for maximizing MBCOMT biosynthesis from *P. pastoris* bioreactor cultures. The model was calibrated with the experiments defined in Table 3.

**Table 3:** List of experiments performed for MBCOMT biosynthesis from *P. pastoris* bioreactor methanol-induced cultures based on CCD and ANN modeling

Experiment number (ANN model iterations)	Input variables level			Output	
	Methanol constant feed flow-rate (mL/L/h)	Induction temperature (°C)	DMSO concentration [%(v/v)]	Observed	Predicted
DoE					
1	1	20	4	126.1	122.2
2	3	20	4	163.9	139.3
3	1	30	4	47.4	97.0
4	3	30	4	188.0	187.1
5	1	20	6	138.0	143.8
6	3	20	6	130.6	139.4
7	1	30	6	151.4	97.5
8	3	30	6	(153.9)	358.1
9	1	25	5	105.3	116.5
10	3	25	5	137.9	134.2
11	2	20	5	115.2	136.8
12	2	30	5	101.1	105.5
13	2	25	4	183.9	197.2
14	2	25	6	222.6	218.5
15	2	25	5	252.5	243.3
16	2	25	5	243.8	243.3
17	2	25	5	230.3	243.3
I					
18	1	22.5	6	364.3	343.1
19	1	22.5	6	364.6	343.1
20	1	22.5	6	357.6	343.1
II					
21	2.9	30	6	390.6	383.1
22	2.9	30	6	391.5	383.1
III					
23	3	30	6	377.1	358.1
24	3	30	6	377.4	358.1
IV					
25	2.5	30	6	263.0	258.9
26	2.5	30	6	283.7	258.9
Final validation					
27	2.9	30	6	–	384.8

The predicted values of MBCOMT specific activity (nmol/h/mg of protein) are those obtained in the last optimization iteration. Observed outputs in parentheses represent the model outliers.

### Modeling of the methanol induction phase using artificial neural network

The ANN model was applied for the optimization of the induction phase for MBCOMT biosynthesis in *P. pastoris* bioreactor cultures using a stepwise process until the maximum MBCOMT biological activity was achieved. Four iterations were required to achieve the maximum MBCOMT specific activity (384.8 nmol/h/mg of protein) under the optimal conditions [30°C, 2.9 mL/L/H methanol constant flow-rate and 6% (v/v) DMSO] and to validate the model with new experiments. In the end, an improvement of 1.53-fold over the best conditions performed in the DoE step (see experiment 15, Table 3) was achieved while an improvement of 6.4-fold over the small-scale biosynthesis in baffled shake-flasks was achieved.

**Fig. 4:**
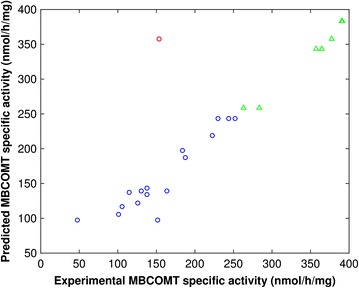
ANN modeling results of MBCOMT specific activity for the last optimization steps. *Blue circle*, *red circles* and *green triangles* represent data from the CCD, outliers and from model optimization.

**Fig. 5:**
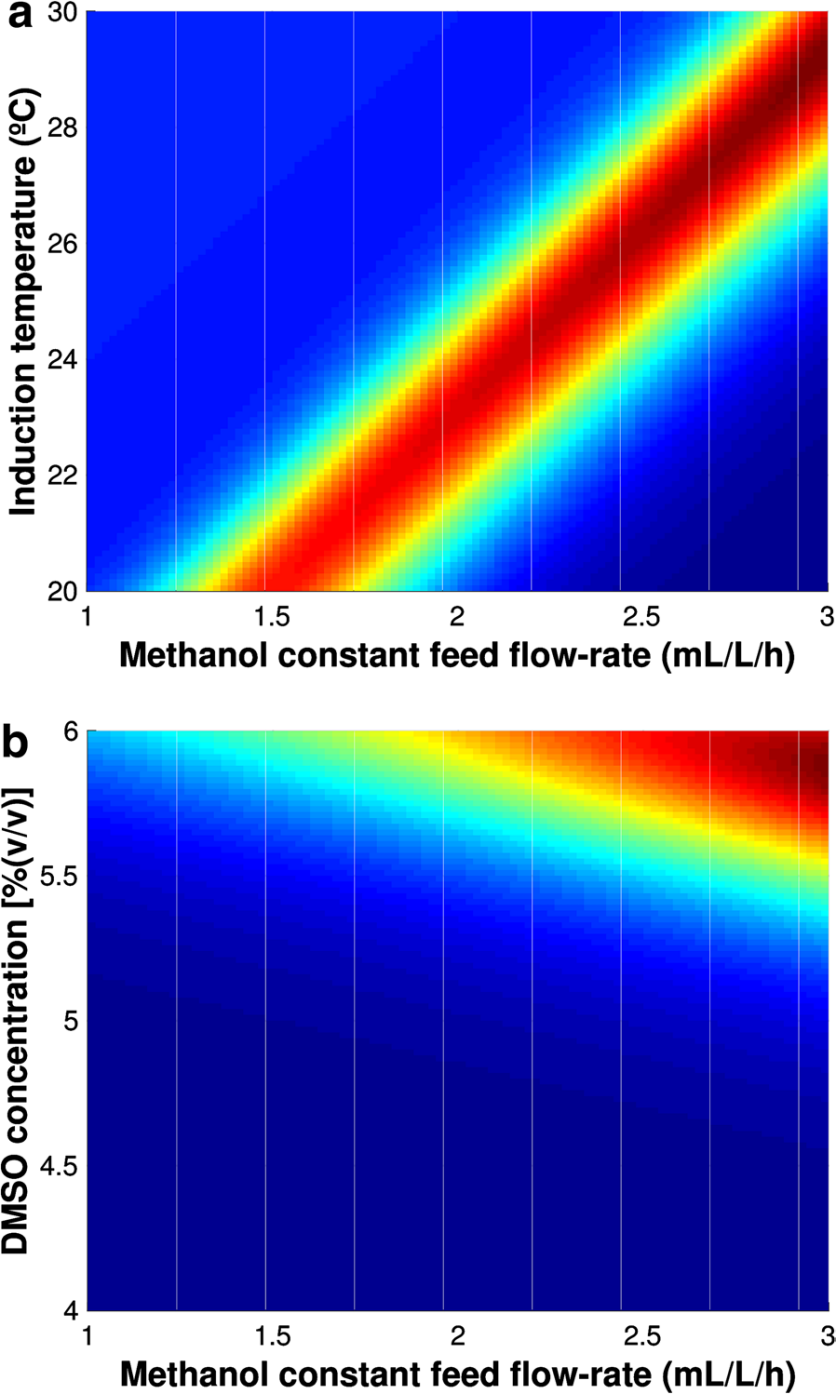
Contour plots of MBCOMT specific activity as function of induction temperature, methanol constant flow-rate and DMSO concentration: **a** modeling results of MBCOMT specific activity as function of DMSO concentration and methanol constant flow-rate for the last optimization step. **b** Modeling results of MBCOMT specific activity as function of induction temperature and methanol constant flow-rate for the last optimization step.

The obtained ANN model is mostly unbiased because the slope and R^2^ of the fitting between the measured and predicted output were close to 1 (0.9064 and 0.97161, respectively) (see Fig. 4). In Fig. 5 are depicted the contour plots obtained from the ANN model for two combinations between the three operational conditions in study. The modeling results showed that the MBCOMT specific activity is sensitive to the operational conditions. The ANN parameters for the final validation model are presented in Additional file 1.

### Bioprocess monitoring at the optimal conditions estimated by the ANN model

**Fig. 6:**
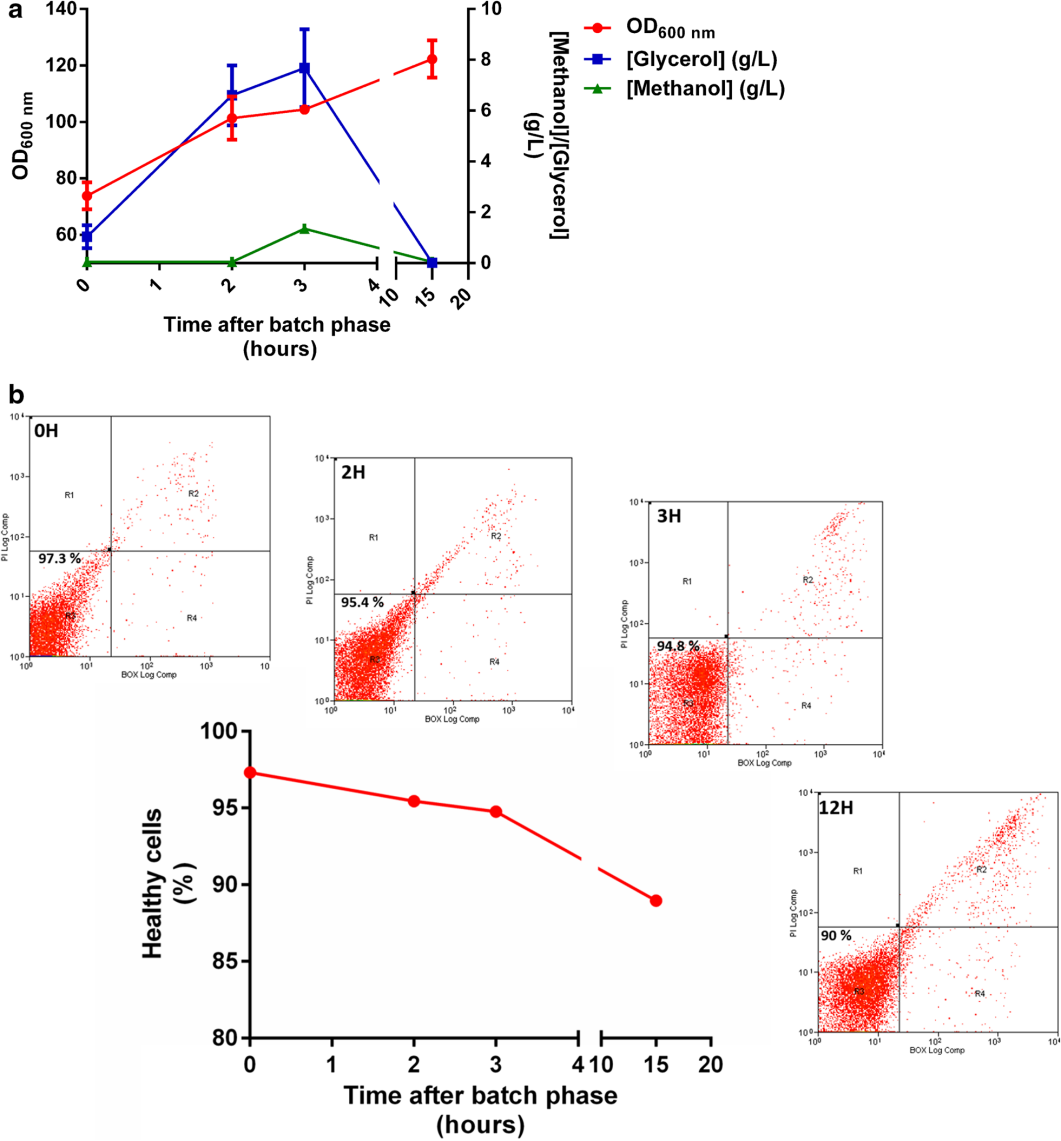
Time course analysis of biomass levels, carbon sources concentrations and number of healthy *P. pastoris* cells at different stages of the optimal point estimated by the ANN model [30°C, 2.9 mL/L/H methanol constant flow-rate and 6% (v/v) DMSO]. **a** Biomass levels measured by spectrophotometric determination at 600 nm and methanol and glycerol levels measurements by HPLC with RID; (each value represents the mean of three independent samples). **b** Dot plots of green fluorescence of cells (BOX, x-axis) plotted against red fluorescence (PI, y-axis) obtained with cell samples taken at different stages of the optimum point retrieved from the ANN modelling. Three main subpopulations of cells can be distinguished corresponding to: healthy cells, no staining; cells with depolarized membranes, stained with BOX; and cells with permeabilized membranes, stained with PI. A total of 10,000 events were collected for these analysis. The variation on the percentage of healthy cells at different stages of the bioprocess is depicted in the *graph*. Each experiment was conducted in duplicate.

At the optimal conditions estimated by the ANN model [30°C, 2.9 mL/L/H methanol constant flow-rate and 6% (v/v) DMSO], the carbon source levels as well as the biomass levels and the number of viable/depolarized cells were analyzed, as depicted in Fig. 6. In what concerns to the *P. pastoris* growth, a marked increase in OD_600_ was detected between the end of the batch phase and the fed-batch growth of glycerol and it keeps increasing until the end of the induction phase with a value near 123 units of OD_600_. The methanol and glycerol levels were quantified using a HPLC with refractive index detection and it was verified that the glycerol concentration also increases during the fed-batch glycerol phase, despite the higher accumulation of biomass during this stage. On the other hand, a low consumption of methanol was verified between the second and the third hours of the glycerol fed-batch phase since we consider that the consumption of glycerol is preferred over the methanol. On the other hand, at the end of the induction phase, almost no methanol was detected since *P. pastoris* cells are oxidizing it all, what can be indicating that the AOX promoter is highly active. Finally, the flow cytometry analysis led us to conclude that the changes introduced at the second hour of the glycerol fed-batch phase (namely the shift to the induction temperature, the addition of DMSO and the initiation of the methanol flow-rate) did not altered significantly the number of viable cells (94.8% compared to 95.4%) in culture. Furthermore, at the end of the induction phase, approximately 90% of viable cells were obtained, a value that is acceptable and is in accordance with *P. pastoris* bioprocesses that include the AOX promoter with a 12 h induction period [39].

To our best knowledge, this is the first systematic study where the interaction between two commonly studied operational parameters (induction temperature and methanol flow rate) and the addition of chemical chaperones (specifically the DMSO) are successfully reported to optimize MP expression by *P. pastoris* bioprocesses using ANN modeling.

## Conclusions

Membrane-bound catechol-*O*-methyltransferase biosynthesis in a highly biological active form was successfully attained for the first time by *P. pastoris* bioreactor cultures under the control of the AOX promoter. The ANN model was able to describe the effects of temperature, DMSO concentration and methanol flow-rate on MBCOMT specific activity, as shown by the good fitness between the predicted and measured values. At the optimal conditions estimated by the ANN model [30°C, 2.9 mL/L/H methanol constant flow-rate and 6% (v/v) DMSO], a 1.58-fold increase was obtained for MBCOMT specific activity (384.8 nmol/h/mg of protein) over the highest value achieved in the experimental design while an improvement of 6.4-fold was found over the small-scale biosynthesis in baffled shake-flasks. Furthermore, in these conditions, almost 90% of viable cells were obtained at the end of the induction phase, indicating that the implemented experimental strategy allowed maintaining the viability of *P. pastoris* cultures. This experimental procedure highlighted the potential of chemical chaperones such as DMSO play to improve the yield of recombinant membrane proteins with a different topology than G-coupled receptors. In addition, this is the first systematic study where the interaction between two commonly studied operational parameters (induction temperature and methanol flow rate) and the addition of chemical chaperones (specifically the DMSO) were successfully reported for the optimization of *P. pastoris* bioprocesses using ANN models. The experimental strategy developed in this work shows that the manipulation of fermentation conditions coupled with the addition of specific molecules can open new perspectives in the optimization of *Pichia pastoris* bioprocesses for recombinant membrane protein biosynthesis.

## Methods

### Materials, strains and media

The easy select expression kit for expression of recombinant proteins using pPICZα in *P. pastoris* and zeocin were obtained from Invitrogen (Carlsbad, CA, USA). Bis-(1,3-dibutylbarbituric acid) trimethine oxonol was acquired from Molecular Probes^®^ (Part of Life technologies; Carlsbad, CA, USA). Yeast nitrogen base (YNB), dithiothreitol, *S*-(5′-adenosyl)-l-methionine, epinephrine (bitartrate salt), deoxyribonuclease (DNase), protease inhibitor cocktail, dl-metanephrine hydrochloride, glass beads (500 µm) and propidium iodide were purchased from Sigma-Aldrich (St. Louis, MO, USA). All chemicals used were of analytical grade, commercially available, and used without further purification.

*E. coli* TOP10F’ was used for DNA manipulations. *E. coli* transformants were selected on low-salt Luria–Bertani plates with 25 µg/mL Zeocin. *P. pastoris* X-33 and KM71H was used for fusion gene expression. YPD and YPDS media [40] were used for routine manipulation of *Pichia* cells. *P. pastoris* transformants were selected on YPDS plates with 200 µg/mL Zeocin. Small-scale fermentations were carried out in BMGH and BMMH media [40]. *P. pastoris* bioreactor cultures were carried out in modified basal salts medium (BSM) [27] with 200 µg/mL zeocin and supplemented with trace metal solution (SMT) [27].

### Small-scale MBCOMT biosynthesis in *Pichia pastoris*

Easy select expression kit for expression of recombinant proteins using pPICZα in *P. pastoris* X33 cells (Invitrogen, Carlsbad, CA, USA) was used for the expression of human MBCOMT in its native form and the process was carried out according to manufacturer’s instructions. Specifically, as the correct membrane protein targeting to the membrane is usually enhanced when secretion signals are used [41], the pPICZα expression vector was employed for expressing MBCOMT expression as it contains the alpha mating factor from *Saccharomyces cerevisiae*. For more details about the construction of the expression vector, please refer to Additional file 2. Subsequently, before conducting the initial trials for MBCOMT biosynthesis at a small-scale, the recombinant plasmid was sequenced in order to confirm the presence of the full sequence of the MBCOMT protein. In fact, after the analysis of the obtained results (Please refer to Additional file 3) concerning the sequencing analysis, it was possible to conclude that the recombinant plasmid contains the full sequence of the MBCOMT protein.

Recombinant hMBCOMT biosynthesis at a small-scale was carried out according to the following protocol [21]: cells containing the expression construct were grown at 30°C in YPD plates. A single colony was inoculated in 50 mL of BMGH medium in 250 mL shake flasks. Cells were grown at 30°C and 250 rpm overnight when the OD_600_ typically reached 6.0. Subsequently, since the inoculation volume was fixed to achieve an initial OD_600_ of 1, an aliquot of the fermentation in the medium BMGH was collected and centrifuged at room temperature during 5 min. After centrifuging the cells and ensuring that all glycerol was removed, the cells were resuspended in the induction medium and added to 500 mL shake-flasks to a total volume of 100 mL. The fermentations were carried out during 120 h at 30°C and 250 rpm, the cells were harvested by centrifugation (1,500×*g*, 10 min, 4°C) and stored frozen at −20°C until use.

### Fed-batch *Pichia pastoris* bioreactor cultures

A single colony was used to inoculate a 100 mL BMGH seed culture in 500 mL shake-flasks and it was grown overnight at 250 rpm and 30°C. This culture was grown to an OD_600_ of 6 and used to inoculate 250 mL of modified basal salts medium (BSM) [26] containing 4.35 mL/L SMT [27] and 200 µg/mL zeocin in a 0.75 L (total volume) bioreactor (Infors HT, Switzerland). The bioreactors were operated with strictly controlled parameters including pH, temperature, airflow, agitation and dissolved oxygen. The pH was set at 4.7 and the DO set point was 20%. The temperature was 28°C in the batch phase while the pH was set at 4.7 during the entire assay and maintained by the addition of 12.5% (v/v) ammonium hydroxide and 0.75 M sulfuric acid. Foaming was controlled manually by the addition of the antifoam agent antifoam A (Sigma-Aldrich, St. Louis, MO, USA). The dissolved oxygen concentration was maintained at 20% by automatic adjustment of the airflow (maximum gas flow-rate used was 2 vvm) and the agitation rate (maximum agitation rate was fixed in 950 rpm). Preliminary trials were carried out in order to determine the best strategy for the biosynthesis of MBCOMT from *P. pastoris*. Therefore, unless otherwise stated, the optimized strategy (see Fig. 3) consisted of a glycerol batch phase that was carried out at 28°C until all glycerol had been consumed, indicated by a DO spike to 45%. Then, a glycerol fed-batch phase was initiated with a constant feed rate of 18.54 mL/L of 50% (v/v) glycerol containing 12 mL/L of SMT during 3 h. After 2 h elapsed, a transition phase was initiated through the addition of a 100% methanol at a constant feed rate, the temperature was changed for the induction temperature and the DMSO was added to the reaction vessel. The constant methanol feed rate, the temperature and the DMSO concentration were defined according to the experimental design. Then, after 3 h elapsed, the induction phase was maintained during additional 12 h using methanol as sole carbon and energy source. The whole system was controlled by IRIS software (Infors HT, Switzerland) and, in particular, the addition of feed medium was achieved using peristaltic pumps that were automatically controlled through a feeding profile previously programmed.

### MBCOMT recuperation

Cell suspensions were broken down using a lysis buffer (150 mM NaCl, 10 mM DTT, 50 mM Tris, 1 mM MgCl_2_, pH 8.0) and freshly made protease inhibitors (1 mM PMSF, 5.0 µg/mL leupeptin and 0.7 µg/mL pepstatin A) containing an equal volume of acid-washed glass beads (500 µm, Sigma-Aldrich, St. Louis, MO, USA). The mixture was vortexed seven times for 1 min with an interval of 1 min on ice and centrifuged at 500*g* (4°C) for 5 min to remove cell debris and glass beads. Finally, the supernatant was collected, DNase (Sigma-Aldrich, St. Louis, MO, USA) (1 mg/mL) was added and the MBCOMT specific activity was determined (see “Determination of copy number by qPCR” for details).

### Experimental design

A CCD with three levels and three factors was employed for the experimental design. The factors and levels for the optimization of MBCOMT specific activity were conditions associated with the fermentation process, namely, the temperature (20, 25 and 30°C), the 100% (v/v) methanol constant feed rate (1, 2 and 3 mL/h/L of culture) and the DMSO concentration [4, 5 and 6% (v/v)]. Table 2 lists the fermentation conditions parameters used in the experimental design and in model development and optimization by ANN.

### Artificial neural network

A feed-forward artificial neural network was applied to predict the MBCOMT specific activity as function of the fermentation conditions (temperature, methanol constant flow-rate and DMSO concentration). The ANN models were implemented in MATLAB™ using the Neural Network Toolbox. The ANN structure included an input layer with three neurons (one for each input variables), an output layer with one neuron (MBCOMT specific activity) and one hidden layer with two neurons (3/2/1). Therefore, the resulting model contains a total of 11 parameters. The transfer functions of the input and output layers, the mathematically representation of the output function and the ANN structure were described elsewhere [18]. The ANN structure was built using the “newff” function. ANN was trained with the Levenberg–Marquardt back-propagation function, up to 1,000 epochs, using the “train” function. The learning rate and the ratio to increase learning rate were set at 0.01 and 1.05, respectively.

### Flow cytometry assays

Cellular viability was assessed during the fermentation runs. Samples were collected at specific periods and analyzed by flow cytometry following the protocol described by Hyka and co-authors [39]. Briefly, the samples OD_600_ was measured, a dilution with PBS buffer was prepared to obtain a final OD_600_ of 0.1 and appropriated volumes of PI and BOX were added in order to attain final concentrations of 10 and 2 mg/L, respectively. The samples were incubated for 15 min at room temperature in the dark, centrifuged for 10 min at 1,500 rpm, resuspended in PBS and sonicated in the “hotspot” during 1 min. The samples were analyzed on a BD Biosciences FACSCalibur (Becton–Dickinson GmbH, Heidelberg, Germany), acquisition was performed with CellQuest™ Pro Software Light scatter measurements and fluorescence was collected in two optical channels, FL1 (515–545 nm, BOX) and FL4 (>670 nm, PI). Threshold was set on SSC to exclude noise, other particles and debris while sample acquisition was operated at flow rate of no more than 300 events per second and a total of 10,000 cells were gated and analyzed in each sample. Data analysis was performed using FCS Express Version 3 Research Edition (De Novo Software™, Los Angeles, CA, USA). The samples were incubated 30 min at 70°C to provide positive staining controls, thereby allowing detection of dead cells and were incubated 2 min at 60°C in order to provide the identification of three subpopulations.

### HPLC analytical methods

The methylating efficiency of recombinant MBCOMT was evaluated by measuring the amount of metanephrine using epinephrine as substrate and as previously described [42]. Briefly, the MBCOMT lysates were incubated at 37°C for 15 min, using epinephrine as substrate and the reaction was stopped with 2 M of perchloric acid. Then, after processing the samples [42], the metanephrine levels in the samples were determined using HPLC with electrochemical detection in a coulometric mode, as previously described [43]. On the other hand, the levels of glycerol and methanol in the culture broth were quantified using a HPLC coupled to a 1260 Infinity Refractive Index Detector (Agilent, Santa Clara, CA, USA), according to what was previously described [21]. The chromatographic separation was achieved on a cation-exchange analytical column Agilent Hi-Plex H (300 × 7.7 mm i. d.; 8 µm) and the analysis was performed at 65°C with a flow rate of 0.6 mL/min using isocratic elution with 0.005 M H_2_SO_4_. The samples were centrifuged at 6,000 rpm for 10 min and the supernatant was filtered prior the injection through a 0.22 µm cellulose-acetate filter.

### Determination of copy number by qPCR

The recombinant gene dosage present in the plasmid pPICZα-hMBCOMT introduced into the strains X33 and KM71H was determined according to the method reported by Nordén and collaborators [23]. Initially, gDNA was extracted from untransformed colonies of X33 and KM71H *P. pastoris* strains as well as from the X33 and KM71H transformants using the Wizard SV Genomic DNA Purification System (Promega, Madison, USA) supplemented with zymolyase. Briefly, for internal standardization, a primer pair—PpAOX2_Prom_FW and PpAOX2_Prom_RV (5′-GACTCTGATGAGGGGCACAT-3′ and 5′-TTGGAAACTCCCAACTGTCC-3′, respectively)—was used that amplifies a stretch of the AOX2 promoter sequence, which is present as one copy in *P. pastoris* genome [23]. Then, in order to determine the number of recombinant gene sequences, it was designed another primer pair—PpAOX1_TT_FW and Pp_AOX1_TT_RV (5′-TGGGCACTTACGAGAAGACC-3′ and 5′-GCAAATGGCATTCTGACATC-3′, respectively)—that is directed towards the 3′TT sequence of the AOX1 gene, which is also present in the pPICZ and also in the pPICZ α plasmids and is integrated together with the gene of interest [23]. The mean efficiency (E) of the two primer pairs was determined according to the serial dilution method using gDNA extracted from both untransformed strains, starting from 100 ng. For each reaction, 10 ng of template were used and the thermal cycler was programmed to perform an initial incubation step at 95°C during 10 min and then 40 cycles of: 15 s at 95°C, 30 s at 60°C, 30 s at 72°C. According to what was previously described by Nordén and collaborators [23], the average copy number was calculated with the following equation:

$$\begin{aligned} Ravg &= \frac{{E^{- \varDelta \varDelta Ct\;sample}}}{{E^{- \varDelta \varDelta Ct\;references}}}\\ &= \;\frac{{E^{- \varDelta Ct\;sample}}}{{E^{- \varDelta Ct\;references}}}\\ &= \frac{{E^{- (Ct\;A\;sample - Ct\;B\;sample)}}}{{E^{- (Ct\;A\;references - Ct\;B\;references)}}} \end{aligned}$$

where Ravg is the average copy number, E the mean primer efficiency, Ct the critical take off cycle, sample the clone in study, reference the strain used (X33 or KM71H), A the AOX1-TT, B the AOX2 promoter. Finally, in order to obtain the MBCOMT copy number, the AOX1 TT copy number was subtracted by 1 to compensate for the endogenous AOX1 TT sequence.

## Additional files

Additional file 1: ANN parameters employed for the final validation model. Additional file 2: Detailed description of the construction of the expression vector pPICZα-hMBCOMT. Additional file 3: Sequencing data of the recombinant expression vector pPICZα-hMBCOMT.

## Abbreviations

ANN: artificial neural network
AOX: alcohol oxidase
BSM: basal salts medium
CCD: central composite design
COMT: catechol-*O*-methyltransferase
DO: dissolved oxygen
DMSO: dimethylsulfoxide
MBCOMT: membrane-bound catechol-*O*-methyltransferase
MP: membrane protein
OD_600_: optical density 600 nm
PI: propidium iodide
*P. pastoris*: *Pichia pastoris*
SAM: *S*-adenosyl-l-methionine

## References

[1] Bawa Z, Bland CE, Bonander N, Nora N, Cartwright SP, Clare M (2011). Understanding the yeast host cell response to recombinant membrane protein production. Biochem Soc Trans.

[2] Bonifacio MJ, Palma PN, Almeida L, Soares-da-Silva P (2007). Catechol-*O*-methyltransferase and its inhibitors in Parkinson’s diseaese. CNS Drug Rev.

[3] Zhu BT (2002). On the mechanism of homocysteine pathophysiology and pathogenesis: a unifying hypothesis. Histol Histopatol.

[4] Zhu BT, Liehr JG (1993). Inhibition of catechol-*O*-methyltransferase catalyzed *O*-methylation of 2- and 4-hydroxyestradiol by catecholamine: implications for the mechanism of estrogen-induced carcinogenesis. Arch Biochem Biophys.

[5] Mattanovich D, Sauer M, Gasser B (2014). Yeast biotechnology: teaching the old dog new tricks. Microb Cell Fact.

[6] Vogl T, Hartner FS, Glieder A (2013). New opportunities by synthetic biology for biopharmaceutical production in *Pichia pastoris*. Curr Opin Biotechnol.

[7] Noseda DG, Blasco M, Recupero M, Galvagno MA (2014). Bioprocess and downstream optimization of recombinant bovine chymosin B in *Pichia* (*Komagataella*) *pastoris* under methanol-inducible AOX1 promoter. Protein Expr Purif.

[8] Darby RA, Cartwright SP, Dilworth MV, Bill RM (2012). Which yeast species shall I choose? *Saccharomyces cerevisiae* versus *Pichia pastoris* (review). Methods Mol Biol.

[9] Batra J, Beri D, Mishra S (2014). Response surface methodology based optimization of beta-glucosidase production from *Pichia pastoris*. Appl Biochem Biotechnol.

[10] Ramon A, Marin M (2011). Advances in the production of membrane proteins in *Pichia pastoris*. Biotechnol J.

[11] Shukla AK, Haase W, Reinhart C, Michel H (2007). Heterologous expression and comparative characterization of the human neuromedin U subtype II receptor using the methylotrophic yeast *Pichia pastoris* and mammalian cells. Int J Biochem Cell B.

[12] Asada H, Uemura T, Yurugi-Kobayashi T, Shiroishi M, Shimamura T, Tsujimoto H (2011). Evaluation of the *Pichia pastoris* expression system for the production of GPCRs for structural analysis. Microb Cell Fact.

[13] Andre N, Cherouati N, Prual C, Steffan T, Zeder-Lutz G, Magnin T (2006). Enhancing functional production of G protein-coupled receptors in *Pichia pastoris* to levels required for structural studies via a single expression screen. Protein Sci.

[14] Cos O, Ramon R, Montesinos JL, Valero F (2006). Operational strategies, monitoring and controlo f heterologous protein production in the methylotrophic yeast *Pichia pastoris* under different promoters: a review. Microb Cell Fact.

[15] Afonso A, Pereira P, Queiroz JA, Sousa A, Sousa F (2014). Purification of Pre-miR 29 by a new *O*-phospho-l-tyrosine affinity chromatographic strategy optimized using design of experiments. J Chromatogr A.

[16] Giordano PC, Martinez HD, Iglesias AA, Beccaria AJ, Goicochea HC (2010). Application of response surface methodology and artificial neural networks for optimization of recombinant Oryza sativa non-symbiotic hemoglobin 1 production by *Escherichia coli* in medium containing byproduct glycerol. Bioresour Technol.

[17] Abdel-Fattah YR, Soliman NA, Yousef SM, El-Helow ER (2012). Application of experimental designs to optimize medium composition for production of thermostable lipase/esterase by *Geobacillus thermodenitrificans* AZ1. Genet Eng Biotechnol J.

[18] Silva R, Ferreira S, Bonifacio MJ, Dias JM, Queiroz JA, Passarinha LA (2012). Optimization of fermentation conditions for the production of human soluble catechol-*O*-methyltransferase by *Escherichia coli* using artificial neural networks. J Biotechnol.

[19] Leonardi D, Salomon CJ, Lamas MC, Olivieri AC (2009). Development of novel formulations for Chagas disease: optimization of benznidazole chitosan microparticles based on artificial neural networks. Int J Pharm.

[20] Almeida AM, Queiroz JA, Sousa F, Sousa A (2015). Optimization of supercoiled HPV-16 E6/E7 plasmid DNA purification with arginine monolith using design of experiments. J Chromatogr B.

[21] Pedro AQ, Oppolzer D, Bonifacio MJ, Maia CJ, Queiroz JA, Passarinha LA (2015). Evaluation of Mut^S^ and Mut^+^*Pichia pastoris* strains for membrane-bound catechol-*O*-methyltransferase biosynthesis. Appl Biochem Biotechnol.

[22] Aw R, Polizzi KM (2013). Can too many copies spoil the broth?. Microb Cell Fact.

[23] Nordén K, Agemark M, Danielson JAH, Alexandersson E, Kjelibom P, Johanson U (2011). Increasing gene dosage greatly enhances recombinant expression of aquaporins in *Pichia pastoris*. BMC Biotechnol.

[24] Pedro AQ, Bonifacio MJ, Queiroz JA, Maia CJ, Passarinha LA (2011). A novel prokaryotic expression system for biosynthesis of membrane-bound catechol-*O*-methyltransferase. J Biotechnol.

[25] Cos O, Serrano A, Montesinos JL, Ferrer P, Cregg JM, Valero F (2005). Combined effect of the methanol utilization (Mut) phenotype and gene dosage on recombinant protein production in *Pichia pastoris* fed-batch cultures. J Biotechnol.

[26] Maurer M, Kuhleitner M, Gasser B, Mattanovich D (2006). Versatile modeling and optimization of fed batch processes for the production of secreted heterologous proteins with *Pichia pastoris*. Microb Cell Fact.

[27] Ferrara MA, Severino BMN, Mansure JJ, Martins AS, Oliveira EMM, Siani AC (2006). Asparaginase production by a recombinant *Pichia pastoris* strain harbouring Saccharomyces cerevisiae ASP3 gene. Enzyme Microb Technol.

[28] Holmes WJ, Darby RA, Wilks MD, Smith R, Bill RM (2009). Developing a scalable model of recombinant protein yield from *Pichia pastoris*: the influence of culture conditions, biomass and induction regime. Microb Cell Fact.

[29] Pla IA, Damasceno LM, Vannelli T, Ritter G, Batt CA, Shuler ML (2006). Evaluation of Mut^+^ and Mut^S^*Pichia pastoris* phenotypes for high level extracellular scFv expression under feedback control of the methanol concentration. Biotechnol Prog.

[30] Xia H, Liu L, Reinhart C, Michel H (2008). Heterologous expression of human Neuromedin U receptor 1 and its subsequent solubilization and purification. Biochim Biophys Acta.

[31] Salunkhe S, Soorapaneni S, Prasad KS, Raiker VA, Padmanabhan S (2010). Strategies to maximize expression of rightly processed human interferon α2b in *Pichia pastoris*. Protein Expr Purif.

[32] Wang Y, Wang Z, Du G, Hua Z, Liu L, Li J (2009). Enhancement of alkaline polygalacturonate lyase production in recombinant *Pichia pastoris* according to the ratio of methanol to cell concentration. Bioresour Technol.

[33] Anasontzis GE, Salazar Penã M, Spadiut O, Brumer H, Olsson L (2014). Effects of temperature and glycerol and methanol-feeding profiles on the production of recombinant galactose oxidase in *Pichia pastoris*. Biotechnol Prog.

[34] Murata Y, Watanabe T, Sato M, Momose Y, Nakahara T, Oka S (2003). Dimethyl sulfoxide exposure facilitates phospholipid biosynthesis and cellular membrane proliferation in yeast cells. J Biol Chem.

[35] Sanmartín-Suárez C, Soto-Otero R, Sánchez-Sellero I, Méndez-Álvarez E (2011). Antioxidant properties of dimethylsulfoxide and its viability as a solvent in the evaluation of neuroprotective antioxidants. J Pharm Toxicol Methods.

[36] Hong F, Meinander NQ, Jonsson LJ (2002). Fermentation strategies for improved heterologous expression of laccase in *Pichia pastoris*. Biotechnol Bioeng.

[37] Wu JM, Wang SY, Fu WC (2012). Lower temperature cultures enlarge the effects of *Vitreoscilla* hemoglobin expression on recombinant *Pichia pastoris*. Int J Mol Sci.

[38] Jahic M, Wallberg F, Bollok M, Garcia P, Enfors SO (2003). Temperature limited fed-bacth technique to control of proteolysis in *Pichia pastoris* bioreactor cultures. Microb Cell Fact.

[39] Hyka P, Zullig T, Ruth C, Looser V, Meier C, Klein J (2010). Combined use of fluorescent dyes and flow cytometry to quantify the physiological state of *Pichia pastoris* during the production of heterologous proteins in high-cell-density fed-batch cultures. Appl Environ Microbiol.

[40] *Pichia* Fermentation Process Guidelines, Version B 053002 (2002) Invitrogen Company (Carlsbad, CA, USA). http://www.invitrogen.com. Assessed 11 Jan 2015

[41] Byrne B (2015). *Pichia pastoris* as an expression host for membrane protein structural biology. Curr Opin Struct Biol.

[42] Passarinha LA, Bonifacio MJ, Queiroz JA (2007). Comparative study on the interaction of recombinant human soluble catechol-*O*-methyltransferase on some hydrophobic adsorbents. Biomed Chromatogr.

[43] Pedro AQ, Soares RF, Oppolzer D, Santos FM, Rocha LA, Gonçalves AM (2014). An improved HPLC method for quantification of metanephrine with coulometric detection. J Chromatogrph Sep Tech.

