# Supplementary material for: An artificial neural network for membrane-bound catechol-O-methyltransferase biosynthesis with Pichia pastoris methanol-induced cultures
Source: Microb Cell Fact. 2015 Aug 7;14:113. doi: 10.1186/s12934-015-0304-7 (PMC4527236; doi:10.1186/s12934-015-0304-7)
Supplement: Additional file 2: — Detailed description of the construction of the expression vector pPICZα-hMBCOMT. [file 12934_2015_304_MOESM2_ESM.docx]

**Additional File 1**

- 1. **Construction of the expression vector:**

Briefly, the DNA fragment coding for MBCOMT was obtained from the pNCMO2_MBCOMT expression vector [23]. Previously constructed by our research group by PCR using specific primers for cloning (forward primer; 5’ AACTCGAGAAAAGAATGCCGGAGGCCCCGCCT 3’; reverse primer, 5’ AACTCGAGTCAGGGCCCTGCTTCGCTGCCTG 3’). PCR was conducted as follows: denaturation at 95 ºC for 5 minutes, followed by 30 cycles at 95 ºC for 30 seconds, 60 ºC for 30 seconds and 72 ºC for one minute, and a final elongation step at 72ºC for 5 minutes. The amplified DNA was purified by low melting agarose gel electrophoresis, digested with Xho I and cloned into the vector pPICZα (previously digested with Xho I) by T4 DNA ligase. This construct was transformed into *E. coli* TOP10F’ cells, grown overnight at 37ºC in plates with low salt luria bertani-agar medium containing zeocin (25 µg/mL) and colonies were screened for the presence of the construct pPICZα-hMBCOMT. Therefore, some colonies were inoculated in 2.0 mL of low salt luria bertani medium and grown at 37ºC and 250 rpm overnight. From these cultures, highly purified plasmids were prepared using Wizard SV Plus SV Minipreps and were then subjected to DNA sequence analysis to confirm the identity of the amplicon, orientation and frame. This was confirmed to correspond to human MBCOMT gene [23]. Then, the target plasmid was introduced into freshly made *P. pastoris* X33 and KM71H competent cells by electroporation according to the manufacturer’s instructions. After plating the resultant mixture in YPDS plates and following the incubation at 30 ºC during 4 days, high level expression transformants were screened and further tested to confirm the methanol utilization phenotype. Finally, the stable occurrence of the expression cassette was verified in the colonies gDNA by PCR using AOX1 promoter and terminator specific primers (AOX1 5’ GACTGGTTCCAATTGACAAGC 3’ and AOX1 5’ CAAATGGCATTCTGACATCC).
