# Supplementary material for: An artificial neural network for membrane-bound catechol-O-methyltransferase biosynthesis with Pichia pastoris methanol-induced cultures
Source: Microb Cell Fact. 2015 Aug 7;14:113. doi: 10.1186/s12934-015-0304-7 (PMC4527236; doi:10.1186/s12934-015-0304-7)
Supplement: Additional file 3: — Sequencing data of the recombinant expression vector pPICZα-hMBCOMT. [file 12934_2015_304_MOESM3_ESM.docx]

1. Recombinant plasmid sequencing using the 5’ AOX1 forward sequencing primer (5’GACTGGTTCCAATTGACAAGC3’)
   1. Raw data obtained:

>A01_PN2+Primer_fw_27.ab1 1100 0 1100 ABI NNNNNNNCGACTTTTACGACACT

TGAGAGATCAAAAAACAACTAATTATTCGAAACGATGAGATTTCCTTCAATTTTTACTGCTGTTTTATTCGCAGC

ATCCTCCGCATTAGCTGCTCCAGTCAACACTACAACAGAAGATGAAACGGCACAAATTCCGGCTGAAGCTGTCAT

CGGTTACTCAGATTTAGAAGGGGATTTCGATGTTGCTGTTTTGCCATTTTCCAACAGCACAAATAACGGGTTATT

GTTTATAAATACTACTATTGCCAGCATTGCTGCTAAAGAAGAAGGGGTATCTCTCGAGAAAAGAATGCCGGAGGC

CCCGCCTCTGCTGTTGGCAGCTGTGTTGCTGGGCCTGGTGCTGCTGGTGGTGCTGCTGCTGCTTCTGAGGCACTG

GGGCTGGGGCCTGTGCCTTATCGGCTGGAACGAGTTCATCCTGCAGCCCATCCACAACCTGCTCATGGGTGACAC

CAAGGAGCAGCGCATCCTGAACCACGTGCTGCAGCATGCGGAGCCCGGGAACGCACAGAGCGTGCTGGAGGCCAT

TGACACCTACTGCGAGCAGAAGGAGTGGGCCATGAACGTGGGCGACAAGAAAGGCAAGATCGTGGACGCCGTGAT

TCAGGAGCACCAGCCCTCCGTGCTGCTGGAGCTGGGGGCCTACTGTGGCTACTCAGCTGTGCGCATGGCCCGCCT

GCTGTCACCAGGGGCGAGGCTGATCACCATCGAGATCAACCCCGACTGTGCCGCCATCACCCAGCGGATGGTGGA

TTTCGCTGGCGTGAAGGACAAGGTCACCCTTGTGGTTGGAGCGTCCCAGGACATCATCCCCCAGCTGAAGAAGAA

GTATGATGTGGACACACTGGGACATGGTCTTCCTCGACCACTGGAAGGACCGGTACCTGCCGGACACGCTTCTCT

TGAAGGAATGTGGCCTGCTGCGGAAGGGGACAGTGCTACTGGCTGACCACGTGATCTGCCCAGGTGCCGCCAGAC

TTCCTAGCACACGTGCCGCGGGAGCAGCTGCTTTGAAGTGCACACACTACCCAATCGTTTCCTGGNATNNNGGAG

NGGTGNACGGCCTGAGANGCATCTNAA

Specifically, the Xho I restriction site is highlighted at yellow (CTCGAG) while the yeast endopeptidase kex2 recognition site (AAAAGA) and the initiation codon (ATG) for the hMBCOMT gene are highlighted, respectively, in red and green.

- 1. Basic local assignment search tool (<http://blast.ncbi.nlm.nih.gov/Blast.cgi>) of the raw data:


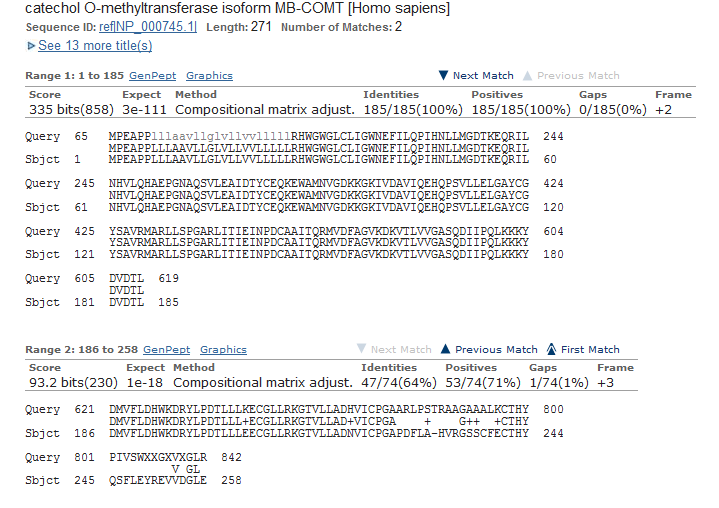


1. Recombinant plasmid sequencing using the 3’ AOX1 reverse sequencing primer (5’ GCAAATGGCATTCTGACATCC 3’)
   1. Raw data obtained:

>B02_PN2+Primer_rv_27.ab1 768 0 768 ABI ANNNNNNNNNANNCAGACCGGTCTT

CTCGTAAGTGCCCAACTTGAACTGAGGAACAGTCATGTCTAAGGCTACAAACTCAATGATGATGATGATGATGGT

CGACGGCGCTATTCAGATCCTCTTCTGAGATGAGTTTTTGTTCTAGAAAGCTGGCGGCCGCCGCGGCTCGAGTCA

GGGCCCTGCTTCGCTGCCTGGGCCCTTGTAGATGGCCTTCTCCAGGCCGTCCACCACCTCCCTGTATTCCAGGAA

CGATTGGTAGTGTGTGCACTCAAAGCAGCTGCTCCCGCGCACGTGTGCTAGGAAGTCTGGCGCACCTGGGCAGAT

CACGTTGTCAGCCAGTAGCACTGTCCCCTTCCGCAGCAGGCCACATTCCTCCAAGAGAAGCGTGTCCGGCAGGTA

CCGGTCCTTCCAGTGGTCGAGGAAGACCATGTCCAGTGTGTCCACATCATACTTCTTCTTCAGCTGGGGGATGAT

GTCCTGGGACGCTCCAACCACAAGGGTGACCTTGTCCTTCACGCCAGCGAAATCCACCATCCGCTGGGTGATGGC

GGCACAGTCGGGGTTGATCTCGATGGTGATCAGCCTCGCCCCTGGTGACAGCAGGCGGGCCATGCGCACAGCTGA

GTAGCCACAGTAGGCCCCCAGCTCCAGCAGCACGGAGGGCTGGTGCTCCTGAATCACGGCGTCCACGATCTTGCC

CTTTCTTGTCGCCCACGTTCATGGCCCACTCCTTCTGCTCCGCAGAATGTGTTCAATGGCCCTCCAGC

Specifically, the Xho I restriction site is highlighted at yellow (CTCGAG) while the stop codon is highlighted in blue.

- 1. Basic local assignment search tool (<http://blast.ncbi.nlm.nih.gov/Blast.cgi>) of the raw data:


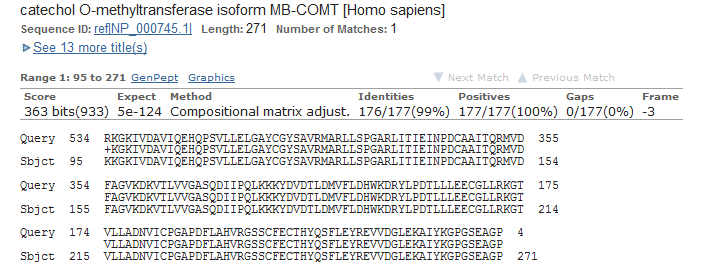


1. Comments:

The recombinant plasmid was sequenced using the AOX forward and reverse sequencing primers. In general, after performing the blast of the raw data obtained, it was observed that the sequence cloned in the recombinant plasmid matches with the nucleotides sequence that codes for human MBCOMT. Moreover, despite some mismatches were obtained at the end of each sequencing reaction, they are attributed to the sequencing reaction itself. Therefore, when the sequencing raw data obtained using the forward and the reverse primers, one can conclude that the cloned sequence obtained in this recombinant plamid codes the human MBCOMT protein.
